# Bexagliflozin as an Adjunct Therapy to Diet and Exercise to Improve Glycaemic Control in Adults with Type 2 Diabetes

**DOI:** 10.17925/EE.2024.20.1.6

**Published:** 2023-12-19

**Authors:** Panagiotis Stachteas, Dimitrios Patoulias, Djordje S Popovic, Polyxeni Athanasiadou, Nikolaos Fragakis

**Affiliations:** 1. Second Department of Cardiology, Aristotle University of Thessaloniki, Thessaloniki, Greece; 2. Outpatient Department of Cardiometabolic Medicine, Second Department of Cardiology, Aristotle University of Thessaloniki, Thessaloniki, Greece; 3. Clinic for Endocrinology, Diabetes and Metabolic Disorders, Clinical Centre of Vojvodina, Medical Faculty, University of Novi Sad, Novi Sad, Serbia; 4. Postgraduate Studies Program in Public Health, Laboratory of Primary Health Care, General Practice and Health Services Research, Aristotle University of Thessaloniki, Thessaloniki, Greece

**Keywords:** Antidiabetic drugs, diet, exercise, glycated haemoglobin, sodium-glucose transporter 2 inhibitors, treatment, type 2 diabetes

## Abstract

Type 2 diabetes (T2D) is one of the leading causes of morbidity and mortality worldwide. Currently, over 10.5% of the adult population has been diagnosed with T2D, and almost 12% of total health expenditure is spent exclusively on T2D management globally. Sodium-glucose cotransporter-2 inhibitors are a relatively new class of oral antidiabetic agents that act by inhibiting renal sodium and glucose reabsorption. Except for their glucose-l owering effects, they have been associated with a more significant weight loss and blood pressure reduction and a lower risk of hypoglycaemia than other commonly prescribed antidiabetic drugs. On 20 January 2023, bexagliflozin became the fifth orally administered sodium-glucose transporter 2 inhibitor to be approved by the US Food and Drug Administration for the treatment of T2D as an adjunct therapy to diet and exercise in the USA after dapagliflozin, canagliflozin, empagliflozin and ertugliflozin. This review aims to discuss the current evidence on the efficacy and safety of bexagliflozin, which provides an important alternative treatment option for patients with T2D.

Type 2 diabetes (T2D) is one of the most common chronic noncommunicable diseases, its incidence is exponentially increasing and is one of the leading causes of morbidity and mortality worldwide.^1^ As of 2021, T2D ranked among the top causes of premature death and was responsible for more than 6.5 million deaths.^2^ Currently, approximately 537 million people (over 10.5% of the adult population) are suffering from T2D^2^; it is expected that the number of adults with T2D will reach 643 million by 2030 and 783 million by 2045.^3^ Notably, almost 12% of total health expenditure is spent exclusively on T2D management globally.^4^ Recently, the coronavirus disease 2019 pandemic significantly disrupted chronic care delivery worldwide and compromised regular medical visits,^5^ with only a partial recovery mediated by the increased telemedicine use.^6^ This situation underlined the unmet need for reducing early morbidity due to nonommunicable diseases, including T2D, a goal that has already been prioritized in the World Health Organization 2030 Agenda for Sustainable Development (Sustainable Development Goals target 3.4).^7^

Sodium-glucose cotransporter-2 (SGLT-2) inhibitors are a relatively new class of oral antidiabetic drugs that inhibit renal sodium and glucose reabsorption by lowering the renal threshold for glucose absorption in the proximal tubule, thereby causing glycosuria and natriuresis.^8^ They reduce plasma glucose concentration in patients with hyperglycaemia but have a small impact on plasma glucose concentrations in euglycaemic individuals.^9^ Furthermore, they have been associated with weight loss, blood pressure reduction and a lower risk of hypoglycaemia than other commonly prescribed antidiabetic drugs, such as insulin and sulfonylureas.^9–11^ In clinical practice, SGLT-2 inhibitors are used as a second-l ine agent for the treatment of T2D after inadequate glycaemic control with metformin or a first-l ine agent for high-risk individuals with cardiovascular disease (CVD), heart failure (HF) or chronic kidney disease (CKD).^12–15^ SGLT-2 inhibitors have received an indication for administration to patients with HF across the spectrum of left ventricular ejection fraction in the recent American College of Cardiology/American Heart Association/Heart Failure Society of America guidelines^16^ because of their benefit in reducing HF hospitalizations and CVD mortality, confirmed by a recent meta-analysis indicating the new fundamental role of SGLT2 inhibitors in the treatment of HF.^17^ In addition to the established CVD benefits, SGLT-2 inhibitors have a renoprotective profile irrespectively of the severity or aetiology of kidney disease and of diabetes status.^18^

In January 2023, bexagliflozin became the fifth orally administered SGLT-2 inhibitor to be approved by the US Food and Drug Administration (FDA) for the treatment of T2D as an adjunct therapy to diet and exercise in the USA, following approval of dapagliflozin, canagliflozin, empagliflozin and ertugliflozin.^19^ The recommended dosage of bexagliflozin is 20 mg once daily in the morning, with or without food. Bexagliflozin is contraindicated in patients with type 1 diabetes, as it may increase the risk of diabetic ketoacidosis, and in patients with end-stage CKD receiving dialysis or those with an estimated glomerular filtration rate (eGFR) of >30 mL/ min/1.73 m^2^.^19^

This narrative review aims to discuss current evidence about the role of the recently FDA-approved SGLT-2 inhibitor bexagliflozin as an adjunct therapy to diet and exercise to improve glycaemic control in adults with T2D. More specifically, the objective of this review is to discuss the clinical data supporting this approval, the mechanism of action of bexagliflozin and how this approval provides an important alternative treatment option for patients suffering from T2D.

## Methods

A narrative literature review was conducted to fill the gap in the international literature on bexagliflozin for the treatment of T2D, and achieve a detailed and comprehensive composition of the limited and quite heterogeneous data on the subject.^20,21^ Three databases (PubMed, Scopus and Google Scholar) were thoroughly screened for articles published up to July 2023. The search terms included “type two diabetes”, “diabetes”, “SGLT2 inhibitors” and “bexagliflozin” combined with the Boolean operators AND, OR and NOT. In addition, snowball sampling, performed by searching reference lists and citation tracking, was implemented in each retrieved article to verify the adequacy of the data. Only studies published in English or Greek were included. Preprint articles published on Medrxiv and SSRN databases, and commentaries, editorials and case reports, were excluded.

The database search produced a large volume of published articles. Duplicate entries were initially found and eliminated, and the title, abstract and keywords of the remaining studies were assessed for their relevance to the study questions. After evaluating the full text of the publications that were pertinent to the subject, a brief critical assessment was followed. Disagreements between the reviewers were discussed and resolved until a consensus was achieved.

## Results

### Mechanism of action: pharmacology

Kidneys play a pivotal role in glucose metabolism, as more than 85% of filtered glucose is reabsorbed, a process intermediated by SGLT-2 located in apical membranes of renal proximal tubules.^22^ In patients with T2D, increased SGLT-2 expression and exertion lead to abnormally elevated glucose reabsorption and, as a result, to persistent high plasma glucose levels.^22–24^ SGLT-2 inhibitors reduce the renal glucose reabsorption capacity by up to 50% of the filtered load.^25^ The decreased reabsorption provokes glucosuria and, consequently, reduces blood glucose concentrations.^26^ Furthermore, the mechanism of action of SGLT-2 inhibitors is independent of the pancreas as β-cell induced the secretion of insulin, making them a significant alternative therapeutic option in patients with T2D.

Bexagliflozin was developed by Theracos, Inc. (Marlborough, MA, USA) and was initially called EGT1442/EGT0001442.^27^ The chemical name of bexagliflozin is (2S,3R,4R,5S,6R)-2-(4-chloro-3-(4-(2-cyclopropoxyethoxy) benzyl)phenyl)-6-(hydroxymethyl)tetrahydro-2H-pyran-3,4,5-triol. The molecular formula of bexagliflozin is C_24_H_29_ClO_7_, and the molecular weight is 464.94 g/mol. Bexagliflozin is a potent (inhibitory constant 2 nmol/L) SGLT-2 inhibitor of benzyl benzene C-glycoside class that is 2,435-fold more selective for SGLT-2, which has elicited a prominent and predictable glucosuria in experimental models, than SGLT-1.^27^ In both healthy individuals and adults with T2D, single and multiple doses of bexagliflozin were associated with dose-dependent elevations in urinary glucose excretion and increases in urine volume.^19^ A 20 mg dose of bexagliflozin delivered near-maximal urinary glucose excretion, with elevated urinary glucose excretion levels maintained with multiple doses.^19^ A single oral dose of bexagliflozin 20 mg is rapidly absorbed, reaching maximum plasma concentration at a median of 2 hours, while the apparent terminal elimination half-l ife of bexagliflozin is approximately 12 hours.^28^ Bexagliflozin is largely metabolized by the UGT1A9 enzyme, which is expressed more highly in the kidneys than the liver, and to a lesser extent, by cytochrome P450 3A to its major metabolite 3'-O-glucuronide.^28^

### Clinical data supporting approval: efficacy data

The FDA recently approved bexagliflozin based on data from a research program assessing its effectiveness and safety that comprised more than 23 clinical studies including more than 5,000 persons with T2D (*Table 1*).^29–37^ According to the results of these trials, bexagliflozin medication lowered glycated haemoglobin (HbA1c) levels compared with placebo. Furthermore, it was shown to be noninferior to glimepiride and sitagliptin. However, bexagliflozin was not found to be superior to placebo in reducing major adverse cardiac events (MACEs), including cardiovascular death, nonfatal myocardial infarction, nonfatal stroke, and hospitalization for unstable angina.^38^

A 12-week, multinational, randomized, double-blind, placebo-controlled, dose-finding phase II study (ClinicalTrials.gov identifier: NCT02390050) evaluated the efficacy and safety of bexagliflozin administered as monotherapy in adults with T2D with an initial HbA1c of between 7.00% and 8.55% who were either treatment naïve or inadequately controlled by one antidiabetic agent and had undergone 6 weeks of treatment abstinence.^39^ Bexagliflozin was found to dose-dependently reduce HbA1c in this population: the decrease in HbA1c measured by the least-squares mean (LSM) from baseline to week 12 was -0.31% in the bexagliflozin 5 mg group, -0.44% in the bexagliflozin 10 mg group, -0.56% in the bexagliflozin 20 mg group and -0.24% in the placebo group. This reduction led to significant placebo-adjusted decreases in HbA1c (the primary endpoint) of -0.55% (95% confidence interval [CI] -0.76%, -0.34%; p<0.0001), -0.68% (95% CI -0.89%, -0.47%; p<0.0001) and -0.80% (95% CI -1.01%, -0.59%; p<0.0001) in the bexagliflozin 5 mg, 10 mg and 20 mg groups, respectively.^39^A statistically significant and clinically meaningful decrease in fasting plasma glucose (FPG) and body mass (secondary endpoints) was also observed in patients receiving 20 mg of bexagliflozin once daily (1.07 mmol L-1 and 1.75 kg, respectively; p<0.0001 for both).^39^

A 24-week, multicentre, randomized, double-blind, placebo-controlled, phase III study (ClinicalTrials.gov identifier: NCT02715258) investigated the safety and efficacy of 20 mg bexagliflozin as monotherapy in patients with T2D and inadequate glycaemic control (HbA1c between 7.0% and 10.5%) with lifestyle interventions (at baseline, bexagliflozin and placebo recipients had a mean HbA1c of 8.1% and 7.9% and a mean FPG level of 169 and 170 mg/dL, respectively).^40^ Monotherapy with oral bexagliflozin 20 mg once daily was found to improve glycaemic control.^40^ At week 24, bexagliflozin significantly decreased HbA1c (the primary endpoint) compared with placebo (adjusted LSM mean change from baseline -0.5% versus. -0.1%; adjusted mean difference from placebo -0.41%, 95% CI -0.66, -0.16; p=0.0012), and the adjusted mean change in FPG levels from baseline was -16 and -3 mg/dL (adjusted mean difference from placebo -14 mg/dL) in the bexagliflozin group and the placebo group, respectively. Systolic blood pressure (SBP) (a secondary outcome) was moderately decreased in patients receiving bexagliflozin, but the change was not statistically significant (-2.14 mmHg; p=0.2340); the same happened with the other secondary outcome, body weight reduction in patients who are overweight or obese (-0.79 kg; p=0.1222).^40^

**Table 1:** Phase II and phase III clinical trials of bexagliflozin on which the recent US Food and Drug Administration approval was based^29–37^

	Bexagliflozin	Comparator	Duration	Phase	Population (N)	ClinicalTrials.gov identifier
1	Monotherapy	placebo	12 weeks	II	292 T2D patients (inadequately controlled)	NCT02390050^29^
2	Monotherapy	placebo	24 weeks	III	207 T2D patients (inadequately controlled)	NCT02715258^30^
3	Monotherapy	placebo	96 weeks	II	288 T2D patients (inadequately controlled)	NCT01377844^31^
4	In combination with metformin	placebo	24 weeks	III	317 T2D patients (inadequately controlled)	NCT03259789^32^
5	Add-on therapy to metformin	glimepiride (Sulphonylurea)	96 weeks	III	426 T2D patients (inadequately controlled)	NCT02769481^33^
6	Add-on therapy to metformin	sitagliptin (DPP-4i)	24 weeks	III	384 T2D patients (inadequately controlled)	NCT03115112^34^
7	Monotherapy or add-on therapy	placebo	24 weeks	III	312 T2D patients with moderate renal impairment (CKD stage 3a/3b)	NCT02836873^35^
8	Add-on therapy to other antidiabetic agents or insulin	placebo	30 months	III	1701 T2D patients with established cardiovascular disease (CVD) or at increased risk for CVD	NCT02558296^36^
9	Add-on therapy to metformin	dapagliflozin	24 weeks	III	390 T2D patients (inadequately controlled)	NCT05159882^37^

*DPP-4 = dipeptidyl peptidase-4.*

In another 96-week, multinational, randomized, double-blind, placebo-controlled, phase II clinical study (ClinicalTrials.gov identifier: NCT01377844) evaluating the safety and effectiveness of bexagliflozin as a monotherapy in patients with T2D and inadequate glycaemic control (HbA1c between 7.0% and 10.0% at baseline), bexagliflozin 20 mg once daily monotherapy was found to be superior to placebo in improving glycaemic control in T2D patients who were either treatment naïve or patients who had been previously treated with antidiabetic therapies but had been instructed to stop taking the drugs at the beginning of the 2-week placebo run-in period.^41^ The placebo-adjusted mean change in HbA1c (the primary outcome) from baseline to week 24 was -0.79% (95% CI -0.53, -1.06 [-5.8, -11.6]; p<.0001). This effect persisted until week 96, at which point the placebo-adjusted decrease in HbA1c was -1.08% (p<0.0001), and the unadjusted mean change from baseline in HbA1c levels was substantially larger in the bexagliflozin group than the control group (-0.55% versus 0.53%).^41^ The secondary endpoints SBP (-6.3 mmHg) and diastolic blood pressure (DBP) (-2.34 mmHg) and body weight were significantly decreased at week 24 with bexagliflozin compared with placebo. In particular, mean (SD) changes in body mass from baseline for the active arm were -2.63 (3.22) kg at week 24 and -2.41 (3.53) kg at week 96; corresponding values for the placebo arm were -0.67 (2.87) kg and 0.45 (3.63) kg, respectively. Intergroup differences at weeks 24 and 96 (-1.96 kg and -2.86 kg; both p<0.0001) were considered clinically meaningful. These long-l asting benefits persisted until week 96, albeit with a modest attenuation.^41^

A 24-week, multicentre, randomized, double-blind, placebo-controlled, phase III trial (ClinicalTrials.gov identifier: NCT03259789) found that glycaemic control was improved by a combined treatment of oral bexagliflozin 20 mg once daily plus metformin in patients with T2D not adequately managed (HbA1c between 7.5% and 10.5%).^42^ At week 24, patients taking bexagliflozin in combination with metformin had a mean change from baseline in HbA1c of -1.09% (95% CI -1.24%, -0.94%) compared with the control group (placebo in combination with metformin) (-0.56%, 95% CI -0.71%, -0.41%). This resulted in a significant placebo plus metformin-adjusted mean change between the treatment and control arms in HbA1c levels (the primary endpoint) of -0.53% (95% CI -0.74%, -0.32%; p<0.0001). In addition, findings from the same study highlighted that the adjusted mean change from baseline in FPG levels was -42 and -20 mg/dL (adjusted mean difference from placebo in combination with metformin -22 mg/dL).^42^

According to a 96-week, multicentre, randomized, double-blind phase III study (ClinicalTrials.gov identifier: NCT02769481), oral bexagliflozin 20 mg once daily in combination with metformin was noninferior compared with glimepiride (sulphonylurea) plus metformin in the adjusted LSM change from baseline (the primary endpoint) in adults with T2D (HbA1c between 7.0% and 10.5%) only treated with metformin (≥1,500 mg/day).^43^ At week 60, the adjusted mean change from baseline in HbA1c was -0.70% in the bexagliflozin/metformin combination group compared with -0.66% in the glimepiride/metformin combination group. The metformin-adjusted LSM difference between groups (the bexagliflozin arm minus the glimepiride arm) was -0.05% (95% CI -0.21%, 0.11%), and the adjusted mean change from baseline in FPG levels was -22 and -14 mg/dL in the two groups (adjusted mean difference from glimepiride plus metformin -8 mg/dL).^43^ At week 96, the between-group difference was -0.21% (95% CI -0.40, -0.02), suggesting that bexagliflozin plus metformin was superior to glimepiride plus metformin.^43^ In summary, bexagliflozin had an equivalent antidiabetic impact to titrated glimepiride and provided an additional cardiometabolic benefit in terms of body weight loss: the study showed a change in body mass from baseline of -4.31 kg (95% CI -5.10, -3.52; p<0.0001) in patients who were overweight or obese, a change in SBP from baseline of -6.53 mmHg (95% CI -10.56, -2.51; p=0.0008) in patients who were hypertensive, and a mean difference in eGFR between arms was 6.05 mL/min/1.73 m^2^, (95% CI 3.24–8.87; p<0.0001).^43^

Another 24-week, multicentre, randomized, double-blind phase III trial (ClinicalTrials.gov identifier: NCT03115112) was conducted among individuals with T2D and inadequate glycaemic control (HbA1c between 7.0 and 11.0%) only with metformin administered orally (≥1,500 mg/day) to compare the safety and effectiveness of bexagliflozin and sitagliptin as adjuncts to metformin.^44^ The study found that antidiabetic treatment with bexagliflozin 20 mg once daily plus metformin was noninferior compared with sitagliptin (a dipeptidyl peptidase-4 inhibitor) in combination with metformin in terms of change in HbA1c levels from baseline to week 24 (the primary endpoint).^44^ At week 24, the estimated mean change in HbA1c levels from baseline was -0.74% (95% CI -0.86%, -0.62%) in the group receiving the bexagliflozin/metformin combination compared with a reduction of -0.82% (95% CI -0.93%, -0.71%) in the sitagliptin plus metformin group (sitagliptin plus metformin-a djusted mean change from baseline 0.08%, 95% CI -0.07%, 0.22%).^44^ Furthermore, the estimated mean change in FPG levels from baseline was higher among patients in the bexagliflozin plus metformin group compared with those in the sitagliptin plus the metformin group (-1.82 mmol/L versus -1.45 mmol/L, with a difference of -0.37 mmol/L [95% CI -0.70, -0.05; p=0.0123]).^44^ In addition, patients with T2D in the bexagliflozin arm with BMI ≥25 kg/m^2^ exhibited a significant reduction in body weight (-3.35 kg) compared with the sitagliptin group (-0.81 kg), with a mean difference of 2.54 kg (95% CI 1.92–3.15; p<0.0001). Furthermore, patients in the bexagliflozin arm exhibited a marginally statistically not-significant sitagliptin-adjusted reduction in SBP of -2.33 mmHg (95% CI -0.05, 4.70; p=0.0276).^44^

A 24-week, multinational, double-blind, placebo-controlled phase III trial (ClinicalTrials.gov identifier: NCT02836873) was conducted among patients with T2D and inadequate glycaemic control (HbA1c between 7.0% and 10.5%) who were either without antidiabetic treatment or currently managed other approved hypoglycaemic drugs and who presented with CKD stage 3a/3b (eGFR 30–59 ml/min/1.73 m^2^) to evaluate the safety and effectiveness of bexagliflozin.^45^ In this study, bexagliflozin 20 mg once daily significantly decreased placebo-adjusted HbA1c (the primary endpoint) by 0.37% (95% CI 0.20–0.54; p<0.001). The subgroups of participants with CKD stage 3a and 3b also experienced placebo-adjusted HbA1c reductions of 0.31% (95% CI 0.09–0.53; p=0.007) and 0.43%, (95% CI 0.16–0.69; p=0.002), respectively. At week 24, more participants in the bexagliflozin arm reached the goal of HbA1c less than 7% FPG compared with the placebo group (34% versus 22%; p=0.007). As in the studies mentioned above, bexagliflozin treatment over 24 weeks resulted in significant reductions in body weight (1.61 kg, 95% CI 1.00–2.22; p<0.001), SBP (3.8 mmHg, 95% CI 0.6–7.1; p=0.02), FPG (0.76 mmol/L; 95% CI 0.26– 1.26; p=0.003) and albuminuria (-20.10%; 95% CI 2.52–34.56; p=0.03) compared with placebo.^45^

Additionally, according to the Bexagliflozin Efficacy and Safety Trial (BEST), a multicentre, randomized, double-blind, placebo-controlled phase III study (ClinicalTrials.gov identifier: NCT02558296) conducted among patients suffering from T2D (HbA1c between 7.5% and 11.0% and eGFR ≥45) and established CVD disease or multiple CVD risk factors, oral bexagliflozin 20 mg once daily showed a placebo-corrected reduction in HbA1c at week 24 (the primary endpoint) of 0.48% (95% CI -0.56, -0.39, p<0.0001).^38^ In addition, patients receiving bexagliflozin demonstrated a mean reduction in SBP of 3.0 mmHg (95% CI -5.5, -0.4, p=0.02) and a body weight reduction of 2.7 kg (95% CI -3.1, -2.2, p<0.0001) compared with placebo. CV death or hospitalization for HF occurred in 48 (4.2%) participants in the bexagliflozin group and in 31 (5.5%) participants in the placebo arm (hazard ratio [HR] 0.74, 95% CI 0.47–1.17) during a mean period of 30 months. MACEs occurred in 90/1133 (7.9%) patients in the bexagliflozin arm and 57/567 (10.1%) in the placebo group (HR 0.79, 95%

CI 0.56–1.09). Bexagliflozin was noninferior compared with the control group in terms of MACEs and hospitalizations for HF.^38^ More studies are needed to investigate whether bexagliflozin has a similar cardioprotective profile to the other commonly prescribed SGLT-2 inhibitors, which play a well-recognized role in reducing MACEs and hospitalization rates.

A recent meta-analysis pooling data from 5 trials with a total of 2,973 patients with T2D investigating the safety of bexagliflozin in treating CVD and its impact on BP found that oral bexagliflozin 20 mg once daily was not associated with elevated risk for MACE compared with placebo (risk ratio 1.18, 95% CI 0.66–2.10; I^2^=0%; p=0.58); however, it resulted in a significant reduction in SBP by 3.44 mmHg (mean difference [MD] -3.44, 95% CI -4.76, -2.12; I^2^=0%; p<0.001) and in DBP by 1.62 mmHg (MD -1.62, 95% CI -2.97, -0.27; I^2^=0%; p=0.02).^46^

A recently published meta-analysis, which included 3,111 patients with T2D from six studies, found that participants in the bexagliflozin arm exhibited a significant and clinically meaningful reduction not only in HbA1c levels (the primary endpoint) (weighted mean difference [WMD] -0.53%, 95% CI -0.75, -0.31) but also in FPG levels (WMD -1.45 mmol/L, 95% CI -2.32, -0.57), in SBP (WMD -4.66 mmHg, 95% CI -6.41, -2.92), in DBP (WMD -2.12 mmHg, 95% CI -3.94, -0.30) and in body weight (WMD -1.61 kg, 95% CI -2.14, -1.07) compared with the control group.^47^ Moreover, more participants in the bexagliflozin arm reached the goal of HbA1c <7% compared with the control group (odds ratio [OR] 1.94, 95% CI 1.36–2.78).^47^

Finally, an on-going multinational, randomized, double-blind, parallel, active-controlled phase III trial (ClinicalTrials.gov identifier: NCT05159882) is being carried out in China among patients with T2D and inadequate glycaemic control (HbA1c between 7.5% and 11.0%) on metformin (≥1,500 mg/day metformin along with lifestyle interventions).^48^ Patients have been randomized to receive either oral bexagliflozin 20 mg or oral dapagliflozin 10 mg once daily. The trial's primary endpoint is the change in HbA1c levels from baseline to week 24, while the secondary endpoints include the change in FPG, body weight and BP from baseline to week 24.^48^ The preliminary findings of this on-going trial are expected to be published in 2023 and are eagerly awaited.

### Adverse events: safety data

In general, oral bexagliflozin 20 mg once daily, administered either as a monotherapy or combined with other hypoglycaemic drugs, was shown to be well tolerated among subjects suffering from T2D in the clinical trials described.^49^ Common side effects were usually similar between bexagliflozin and active or placebo comparator treatment arms.^28^ The most common side effects reported were increased urination, genital mycotic infections, urinary tract infections and volume depletion phenomena. Similar findings have been attributed to SGLT-2 inhibitors due to glycosuria.^11^ Mild volume depletion (presented with polyuria, dehydration, dizziness, vertigo, presyncope, thirst and, rarely, orthostatic hypotension), weight loss, reduction in SBP and elevation of serum creatinine concentration can also be attributed to the pharmacology of the SGLT2 inhibitors class, which cause osmotic diuresis, natriuresis and caloric wasting.^11,49^

According to a pooled analysis of data from 3 clinical trials,^19^ the most commonly observed adverse effects were increased urination (7% of participants in the bexagliflozin group versus 3% of participants in the placebo group), urinary tract infection (6% of the participants in the bexagliflozin group versus 4% of participants in the control group), female genital mycotic infections (6% of participants in the bexagliflozin group versus 0% of participants in the placebo group), male genital mycotic infection (2% of participants in the bexagliflozin group versus 1% of participants in the control group), thirst (3% of participants in the bexagliflozin group versus 2% of participants in the control group), vaginal pruritus (3% of participants in the bexagliflozin group versus 0% of patients in the placebo arm) and hypoglycaemia (2% of participants in the bexagliflozin group versus 1% of patients in the control group).^49^ According to data from the BEST trial, the most commonly observed adverse effects in patients suffering from T2D and who were at elevated CVD risk were, in general, consistent with other bexagliflozin trials.^38^

Ketoacidosis, a serious, life-threatening condition requiring emergency hospitalization, has been reported in patients with T2D to whom SGLT2 inhibitors (including bexagliflozin) were administered.^19^ Adults suffering from T2D who receive bexagliflozin and show clinical signs and symptoms consistent with dehydration and severe metabolic acidosis (e.g. nausea, vomiting, abdominal pain, general malaise and shortness of breath) should be screened for ketoacidosis irrespectively of blood glucose levels, as it has been observed that, although rarely, bexagliflozin-associated ketoacidosis may be present even when blood glucose is below 250 mg/dL.^11^

In the BEST trial, 5.4 versus 1.4 major fracture events (including hip and femur fractures) occurred per 1,000 patient–years of follow-up among patients participating in the bexagliflozin plus metformin arm compared with placebo plus metformin arm, respectively.^38^ An imbalance in serious fractures was observed during the first 24 weeks of treatment and continued until the termination of the study. Nontraumatic lower limb amputation events were observed among participants in both groups (bexagliflozin plus metformin and placebo plus metformin), and the incidence rates were not statistically significant (8.3 versus 5.1 per 1,000 patient–years; HR 1.64, 95% CI 0.70–3.82).^38^

In a recent meta-analysis, there were no significant differences between patients receiving bexagliflozin and those receiving placebo in the reported adverse outcomes, such as hypoglycaemia (OR 0.95, 95% CI 0.80–1.14; I^2^=0%; p=0.60), genital mycotic infection (OR 3.11, 95% CI 0.86–11.29; I^2^=7%; p=0.08), urinary tract infection (OR 1.04, 95% CI 0.80– 1.36; I^2^=19%; p=0.75), polyuria (OR 1.57, 95% CI 0.83–2.99; I^2^=0%; p=0.17), diabetic ketoacidosis (OR 0.44, 95% CI 0.01–17.59; I^2^=65%; p=0.66) and all-cause mortality (OR 0.76, 95% CI 0.47–1.24; I^2^=0%; p=0.28).^47^ In other clinical studies investigating SGLT-2 inhibitors in patients with T2D, the risk of hypoglycaemia was similarly rare, except in the case of the co-administration of sulfonylureas or insulin, due to the selectivity of these drugs, the preservation of SGLT-1 function, the decreased insulin secretion and the increased glucagon levels.^50^

### Comparison with other sodium-glucose cotransporter-2 inhibitors

The findings of our review are in concordance with a recent meta-analysis investigating the efficacy and safety of bexagliflozin.^47^ They also suggest that bexagliflozin significantly impacts glycaemic management, thus reflecting the existing body of research on other commonly prescribed SGLT-2 inhibitors.^51,52^ Indeed, all SGLT-2 inhibitors have demonstrated comparable outcomes in terms of decreasing HbA1c levels in patients with T2D thus far. An analysis conducted by Shyangdan et al. revealed that individuals receiving SGLT-2 inhibitors had a higher likelihood of attaining a HbA1c level of below 7% than those receiving placebo.^53^ Additionally, a meta-analysis juxtaposing canagliflozin, dapagliflozin or empagliflozin against placebo indicated that all SGLT-2 inhibitors reduced both HbA1c and FPG levels.^54^ In addition, the SGLT-2 inhibitors have consistently been demonstrated to decrease body weight and SBP.^51,55,56^ Our assessment indicates that these outcomes are similarly applicable to bexagliflozin, a more recent addition to this drug category.

T2D is an established risk factor for atherosclerotic CVD and HF.^57^ The effectiveness of SGLT-2 inhibitors in mitigating cardiovascular outcomes, particularly HF incidents, in this population group has been demonstrated consistently.^57^ The EMPA-REG OUTCOME trial (ClinicalTrials.gov identifier: NCT01131676) showed that empagliflozin reduces the rates of cardiovascular-related death, nonfatal myocardial infarction nonfatal stroke and overall mortality compared with placebo among patients with T2D at high risk of cardiovascular events.^56^ Similar trends were observed in the Canagliflozin Cardiovascular Assessment Study (CANVAS) involving canagliflozin.^55^ A recent meta-analysis assessing the efficacy of bexagliflozin for the treatment of patients with T2D identified only two MACEs.^47^ Notably, levels of all-cause mortality did not significantly vary between participants receiving bexagliflozin and those receiving placebo. A meta-analysis assessing the cardiovascular safety of bexagliflozin in a smaller patient cohort also revealed no significant distinction in MACEs between bexagliflozin and placebo.^46^ However, it is important to interpret the results of this meta-analysis cautiously, as the patient count in the bexagliflozin group is considerably smaller than the one included in the CANVAS and EMPA-REG trials.^55,56^

Studies have recorded a limited number of undesirable outcomes associated with SGLT-2 inhibitors, including hypoglycaemia, infections in the genital and urinary tract, fungal infections in the genital area, hypersensitive skin reactions, reduced blood volume, painful urination and diabetic ketoacidosis.^58^ Bexagliflozin was shown to be well tolerated, and it presented an acceptable safety profile with minimal adverse effects and a low risk of hypoglycaemia. The occurrence of hypoglycaemia was not found to be statistically significant in previous meta-analyses investigating empagliflozin and dapagliflozin.^59^ Nonetheless, certain studies have indicated an elevated likelihood of hypoglycaemia in connection with SGLT-2 inhibitors compared with bexagliflozin.^51,52,54,55,60^ Genital fungal infections and amputations have been highlighted as notable adverse outcomes of SGLT-2 inhibitors.^58^ The CANVAS trial showed canagliflozin to be associated with a notable rise in amputation risk.^55^ However, a meta-analysis focusing solely on amputation risk in connection with SGLT2 inhibitors indicated no significant correlation.^61^ Another recent meta-analysis found that bexagliflozin and placebo did not significantly differ in this regard.^47^

Of course, it has to be emphasized that there has not been any solid evidence to support the comparative effectiveness and safety between bexagliflozin and other commercially available SGLT-2 inhibitors. Indeed, no randomized controlled trial directly comparing bexagliflozin with another SGLT-2 inhibitor in the setting of T2D has been published so far. Therefore, whether bexagliflozin is superior or inferior to other SGLT-2 inhibitors that are widely used in daily clinical practice needs to be confirmed. In addition, cost-effectiveness analyses would be useful in the future to document whether bexagliflozin is more cost-effective than other SGLT-2 inhibitors for the treatment of T2D. Based on the available evidence, bexagliflozin appears to be a safe and effective treatment for T2D; however, there is no high-quality evidence supporting the use of bexagliflozin over other SGLT-2 inhibitors in clinical practice. More data are needed in this regard.

## Conclusions

The FDA recently approved bexagliflozin as an adjunct hypoglycaemic therapy to lifestyle interventions such as diet and exercise to improve glycaemic control in patients with T2D. It is an orally administered, highly selective SGLT-2 inhibitor that confers comparable dose-dependent reductions in HbA1c and FPG levels to widely prescribed antidiabetic drugs. As well as glycaemic control, bexagliflozin successfully reduced body weight and BP levels in most phase II/III clinical trials. Furthermore, it seems to be consistently effective and safe for adults with CKD stage IIIa/b. Bexagliflozin has shown to be well tolerated and to have an acceptable safety profile with minimal adverse effects and a low risk of hypoglycaemia. Notably, bexagliflozin demonstrated a safe cardiovascular profile in T2D, as it was noninferior to placebo for MACE in high-risk adults with CVD comorbidities. However, there is still no solid evidence supporting the superiority of bexagliflozin to other commercially available SGLT-2 inhibitors and, thus, prioritizing its use for the treatment of T2D.
